# Perspectives and experiences of compassion in long-term care facilities within Canada: a qualitative study of patients, family members and health care providers

**DOI:** 10.1186/s12877-019-1135-x

**Published:** 2019-05-06

**Authors:** Lorraine Smith-MacDonald, Lorraine Venturato, Paulette Hunter, Sharon Kaasalainen, Tamara Sussman, Lynn McCleary, Genevieve Thompson, Abigail Wickson-Griffiths, Shane Sinclair

**Affiliations:** 1 Faculty of Nursing, University of Calgary, 2500 University Drive NW, Calgary, AB T2N 1N4 Canada; 2 Division of Palliative Medicine, Department of Oncology, Cumming School of Medicine, University of Calgary, 2500 University Drive NW, Calgary, AB T2N 1N4 Canada; 3 Dementia and Cognitive Impairment NeuroTeam, Hotchkiss Brain Institute, University of Calgary, 2500 University Drive NW, Calgary, AB T2N 1N4 Canada; 4 St. Thomas More College, University of Saskatchewan, 1437 College Drive, Saskatoon, SK S7N 0W6 Canada; 5 School of Nursing, McMaster University, 1280 Main Street West, Hamilton, ON L8S 4K1 Canada; 6 School of Social Work, McGill University, 845 Sherbrooke Street West, Montreal, QC H3A 0G4 Canada; 7 Department of Nursing, Brock University, 1812 Sir Isaac Brock Way, St. Catharines, ON L2S 3A1 Canada; 8 College of Nursing, Max Rady Faculty of Health Sciences, University of Manitoba, Helen Glass Centre for Nursing, 89 Curry Place, Winnipeg, MB R3T 2N2 Canada; 9 Faculty of Nursing, University of Regina, 3737 Wascana Parkway, Regina, SK S4S 0A2 Canada; 10 Compassion Research Lab, Faculty of Nursing, University of Calgary, 2500 University Drive NW, Calgary, AB T2N1N4 Canada

**Keywords:** Compassion, Compassionate care, Long-term care, Palliative care, Person-centered care, End-of-life

## Abstract

**Introduction:**

This paper details a subset of the findings from a participatory action research project exploring a palliative intervention in long-term care sites across Canada. The findings presented in this paper relate to understanding compassion within the context of a palliative approach to long-term care.

**Methods:**

Findings presented are drawn from qualitative interviews and focus groups with residents, family members, healthcare providers, and managers from 4 long-term care sites across 4 provinces in Canada. In total, there were 117 individuals (20 residents, 16 family members, 72 healthcare providers, and 9 managers) who participated in one of 19 focus groups. Data was analyzed by multiple members of the research team in accordance with thematic analysis. Individual concepts were organized into themes across the different focus groups and the results were used to build a conceptual understanding of compassion within Long Term Care .

**Findings:**

Two themes, each comprised of 5 sub-themes, emerged from the data. The first theme 'Conceptualizing Compassion in Long-Term Care generated a multidimensional understanding of compassion that was congruent with previous theoretical models. 'Organizational Compassion: resources and staffing', the second major theme, focused on the operationalization of compassion within the practice setting and organizational culture. Organizational Compassion subthemes focused on how compassion could support staff to enact care for the residents, the families, one another, and at times, recognizing their pain and supporting it through grief and mourning.

**Conclusions:**

Results suggest that compassion is an essential part of care and relationships within long-term care, though it is shaped by personal and professional relational aspects of care and bound by organizational and systemic issues. Findings suggest that compassion may be an under-recognised, but essential element in meeting the promise of person-centred care within long-term care environments.

**Electronic supplementary material:**

The online version of this article (10.1186/s12877-019-1135-x) contains supplementary material, which is available to authorized users.

## Background

As baby boomers hit 65 years of age and birthrates continue to decline, Canada, for the first time in its census history, has more adults aged 65 years and over (5.9 M) than it does children under 15 years of age (5.8 M) [1]. Within Canada, long-term care (LTC) plays an increasingly important role in the continuum of care for older adults as people live longer and with more chronic health conditions. These facilities, sometimes also known as nursing homes, provide continuous on-site professional nursing services and personal care assistance for moderate to extensive chronic conditions for predominantly older adults. Healthcare conditions can vary, but cognitive impairments are becoming increasingly more prominent with approximately two out of three LTC residents being diagnosed with Alzheimer’s disease or another dementia, and over 90% of residents in LTC having some form of cognitive impairment [2]. In response to the challenges associated with these conditions, LTC facilities are shifting from traditional medical models to more humanistic approachs to care [3]. Person-centered care within LTC has been described as moving away from caring for residents according to set schedules and routines and instead caring for residents according to their individual lifetime practices, habits, and preferences [4, 5]. Key elements of patient-centered care within LTC are facilitated contact with family, privacy, having choices of food and bathing times, spiritual well-being, and being treated with respect and dignity [6, 7].

Increasingly, integrated models of palliative care, whereby palliative care approaches and services are embedded early in the trajectory of a life-limiting illness versus being confined to the end-of-life or a separate service, are being utilized and recognized as best practice, including in LTC were the majority of residents will die [8, 9]. Palliative care is an approach to care that focuses on improving the quality of life of patients and families who are facing a life-limiting and incurable health condition by addressing physical, social, emotional, and spiritual needs [10]. Dying in LTC is often complicated by complex trajectories associated with multiple co-morbidities and cognitive changes, resource constraints, limited access to physicians, over/under medicating and polypharmacy issues, a lack of training or experience specific to palliative or end-of-life issues, and workload issues [11]. Despite this, place of death is considered an important indicator of quality end-of-life care, with most people reporting a preference to die in their usual place of residence, including LTC [12]. Strengthening a palliative approach can help integrate psychosocial, relational, and spiritual aspects of care, alongside physical care, without requiring LTC facilities to have separate palliative care units [9, 13]. Enhancing quality end-of-life care requires competent and timely advanced care planning, excellent communication, a patient-centered multidisciplinary approach, and compassionate care [14].

Compassionate care, while being a tenant of patient-centered care, is a distinct concept and approach to caring. Compassion, is a core value in healthcare, and is one of the key components of the concept of “care” [15]. It is thus a central tenet of quality healthcare, where it is evident in best practice guidelines, health care reforms, care standards, patient code of rights, and professional organizations’ codes of ethics [16–23]. Etymologically, compassion means “suffering with” and it has been defined as “a deep awareness of the suffering of another coupled with the wish to relieve it” [24–26]. While compassion and care have long been considered hallmarks of healthcare practice, there is some confusion over definitions, with compassion being conflated with related concepts of sympathy and empathy [27, 28]. While empathy and sympathy may be considered part of the conditions for caring, compassion “impels and empowers people to not only acknowledge, but also act” [29]. Patients and family members consistently identify aspects of compassion, for example person-centered and responsive interactions, as indicators of quality care [30, 31].

Although compassionate care seems intuitive, and the majority of clinicians are dedicated to imbuing their practice with compassion, observational studies of healthcare providers and patient reports suggest translating theory into practice is a persistent challenge [31–35]. Despite a growing body of literature exploring the concept of compassion and compassionate care in healthcare in general there has been very little consideration of compassion in the LTC literature. VanderCingel and colleagues conducted a qualitative study of nurses and patients in LTC, identifying seven dimensions of compassion: attentiveness, listening, confronting, involvement, helping presence and understanding—with the study authors recommending that further research is needed to confirm these findings [36]. While a further understanding of healthcare provider and residents’ understandings of compassion is needed, administrators and residents, and family members bring an important perspective that needs to also be integrated.

The findings presented in this paper are a subset of large study, *Supporting a Palliative Approach in Long Term Care (SPA-LTC),* being undertaken across four provinces in Canada. The larger study aims to implement and evaluate an evidence-informed palliative approach in LTC. The purpose of this focused analysis was: (1) to understand and explore perceptions about compassion in the delivery of palliative care from the perspective of residents in LTC, their family members, and healthcare providers (HCPs); and (2) to identify potential facilitators and barriers associated with providing compassionate care to residents in LTC across Canada.

## Method

### Setting & sample

The study was conducted in four LTC sites across Ontario, Manitoba, Saskatchewan, and Alberta, Canada. Sites varied in size, ranging from 50 to 284 residents per site, and varied in ownership, with three of the sites being ‘for-profit’ and one being ‘not-for-profit’. Participants were recruited in the spring of 2015. Staff and manager participants were recruited via information sessions at staff meetings and flyers on staff notice boards, while resident and family participants were initially informed about the study by site staff, and if they expressed interest in learning more about the study were provided further details by the research team. Participants were provided the opportunity to have their questions about participating in the study answered prior to consenting to participate, with research staff reminding participants that participation was completely voluntary and they could withdraw from the study at any time. Written consent was provided by all participants prior to beginning the focus groups. All identifying information was also removed and anonymized during the transcription process to maintain confidentiality. Ethical approval for the study was granted by multiple University Research Ethics Boards (Hamilton Integrated Research Ethics Board #0427; Brock University #15–102; McGill University #281–1214; University of Saskatchewan #15–270; University of Regina #15–190; University of Manitoba #H2015:374; University of Calgary #15–2277) and local health authorities and organizations as required.

### Data collection

Data was collected via focus groups by two members of the research team in each province at participating sites with residents, family members, healthcare providers and managers using a semi-structured interview guide (Additional file 1), which was modified depending on whether the focus group was comprised of residents, family members, healthcare providers or managers. Focus groups were chosen for both pragmatic reasons (scheduling and time constraints) and to allow for rich dialogue between individuals with similar experiences but differing perspectives. Focus groups consisted of between 3 and 8 study participants and were on average 60 min in duration, occurring in a private room within each care facility. There was a total of 19 focus groups, yielding a total sample size of 117 participants (20 residents, 16 family members, 72 healthcare providers, and 9 managers) with each focus group being comprised of similar individuals (i.e. patients, family members, healthcare staff, or manager). Residents, including those with mild cognitive impairment, were purposely included as a participant group, as this subpopulation is of often unduly excluded from research. Patients with significant cognitive impairment, as determined by their health care team, were excluded from this study. Focus groups were digitally recorded and transcribed verbatim by a professional transcriptionist.

### Data analysis

Focus groups were subjected to thematic analysis [37]. Braun and Clarke [37] described thematic analysis as a method for identifying, analyzing, and reporting patterns (themes) in rich detail, which when done properly may also allow for the research to interpret various aspects of the topic. Practically, thematic analysis involves examining the text in detail to identify recurring patterns (open coding) which are refined into ‘themes’ through inductive and/or deductive analysis. That is, those that arise directly from the data and those which relate to theory and previous findings, respectively [37]. For this study, both inductive and deductive processes were used. In coding and generating sub-themes within the theme of *“'Conceptualizing Compassion in LTC'”* it became apparent that findings were congruent with the patient and healthcare provider compassion models previously published [38]. An audit trail was also created by the lead author (LSM) to maintain credibility and rigor throughout the data analysis process by allowing research team members to review and examine each decision and step within the data analysis process [39].

Data analysis began by the research team members (LSM, LV, SS) reading through all the focus group interviews, and, the lead (LSM) and corresponding author (SS) developing preliminary open codes. Once these codes were identified and validated by the third member of the research team (LV), the lead author began combining these codes into the preliminary themes. These preliminary themes were then analyzed and verified by the larger research team as an extra measure of rigor, with any differences being resolved through ongoing discussion between all authors. Having established the preliminary themes, the lead author then wrote an initial draft of the proposed thematic framework, which underwent a secondary round of collective analysis, with the preliminary themes being modified to reflect a more nuanced and rich understanding of the data. While unanticipated, it became apparent at this stage that the sub-themes within the theme of '*Conceptualizing Compassion* in LTC' were congruent with the key domains of the patient and healthcare provider model [38, 40]. Participant quotes, illustrating each sub-theme were selected for their representativeness and incidences of divergent opinions.

## Results

The overall focus of this analysis was to determine how compassion is understood and enacted within LTC facilities within Canada. Direct quotes from various focus group participants have been included to provide evidence and support for the themes and subthemes. While there was slight variance between the different groups, similar themes emerged across all groups, indicating that, while there are differences based on participants’ experiences of their role (i.e. being a resident, family member, healthcare provider), there was consensus on the nature of compassion and its key components. Within this study two main themes, with associated sub-themes, emerged: 1. *Conceptualizing Compassion in LTC*; and 2. *Organizational Compassion: resources and staffing* (Table 1).

**Table 1:** Themes of compassion within LTC

Compassion		
Theme	Conceptualizing Compassion in LTC	Organizational Compassion: resources and staffing
Subtheme	*Virtues*	*Compassion training*
	*Virtuous response*	*Mandated compassion*
	*Seeking to understand*	*Time*
	*Relational communicating*	*Inter-professional compassion*
	*Attending to needs*	*Death, grief, and mourning*

### Theme: Conceptualizing Compassion in LTC

The first theme that emerged from the data related to participants’ perspectives of the nature of compassion within a LTC setting. Five inter-related themes, emerged from the data, illustrating participants’ beliefs that compassion was a multi-dimensional, relational and dynamic construct that was expressed through the innate and embodied virtues (*Virtues* and *Virtuous response)*; a desire to understand a person and their needs *(Seeking to understand)*, verbal and non-verbal communication (*Relational communicating)* and action aimed at addressing a person’s needs (*Attending to needs).*

#### Virtues

Participants initially struggled to clearly articulate what compassion was, but were quickly able to discuss the motivators of compassion as virtues. While there were many virtues that participants felt were the antecedents to compassion, the most commonly associated with compassionate care in LTC were: honesty, love, patience, gentleness, kindness, genuineness (genuine concern), understanding, peacefulness, respect, and dignity.

*“Compassion is love … ” (Resident, Focus Group #2);*

*“Friendship, comfort, loving, concerned” (Resident, Focus Group #4)*

*“Compassion is to be kind and gentle and to do our best to respect their wishes” (Healthcare Provider, Focus Group #6)*

*“Compassion is just having it in your heart, the true feelings that you care and showing care and love and respect to whoever is around in that situation” (Healthcare Provider, Focus Group #10)*

Participants across all groups, felt that these virtues were innate within healthcare providers (HCPs) and that residents could sense whether their HCPs possessed these virtues, noting that it was not something that could be “faked”. Residents described this most starkly when compassion was felt to be absent, as without these virtues, their relationship with the HCPs was perceived to be less positive, and their confidence in their HCP’s capacity to demonstrate compassion was diminished.

#### Virtuous response

The second subtheme identified was a *“'virtuous response'”*. This category focused on the initial response of the HCPs in recognizing a resident or family members' need, where their innate virtues were enacted through a desire and initial response to attend to a person’s needs, resulted in a quieter, calmer, more conscientious approach to offering care. When HCPs described what happened to them when they entered this stance, they expressed a deep sense of self-awareness that allowed them to notice and be more fully present to a resident’s need. Practically, HCPs spoke of how, when they found themselves responding in this manner, the focus of their work became less “task-oriented” and became more “person-orientated”; even if they were doing the same tasks.

*“Sometimes it’s in the approach, how we touch someone, whether we are in a hurry or just be gentle” (Managers, Focus Group #1)*

Participants described this experience as being an internal “slow down”, and a move from a “doing stance” into a “being stance”.

*“Because it’s [compassion] not necessarily a task. It’s not a what, it is a how [you care]” (Manager, Focus Group #1)*

*“I think it requires if you can take the moment to just flip from your head to your heart … I mean you can be very much working from the heart when you are doing those tasks, which I’m sure everybody here is, but it’s that conscious, mindfulness of going to that place of respect and caring” (Healthcare Provider, Focus Group #4,)*

Residents also expressed an ability to recognize when a HCP response stemmed from their innate virtues, as their demeanor and behaviors took on another dimension that they described as a peaceful, comforting presence, that resulted in them being more willing to understand the residents needs versus their own needs or the needs of the organization.

#### Seeking to understand

The third subtheme further externalized the innate virtues of HCPs within a relationship that was focused on developing a deeper understanding of the person in need. This desire to understand was not confined to cognitive or situational understanding (e.g. of their medical condition), but was highly inter- and intra-personal, requiring HCPs to tap into their own subjective experiences of love, care, and/or suffering, with many HCPs asking themselves ‘how would I like to be treated if the healthcare provider-resident roles were reversed’—referred to by a number of participants metaphorically as the ‘Golden Rule’.

*“Try to understand what they’re going through and feel like it’s your family member and what would you want to be done?” (Healthcare Provider, Focus Group #8)*

Interestingly, one resident also raised this idea by commenting that they tried to reciprocate the compassion that their HCPs gave to them, conjuring images of a compassion feedback loop among other focus group participants. However, the predominant focus in this subtheme was on HCPs seeking to understand a resident’s experience, and recognition of the “humanness” of the resident in their care. HCPs described this as a challenge, particularly within a patient population where cognitive impairment often resulted in challenging behaviors or changes in personality. The majority of HCPs sought to overcome this challenge by trying to acknowledge the personhood and dignity of each resident, regardless of their cognitive status or current behavior, illustrating the largely unconditional nature of compassion in this process.

*“Compassionate care I think is to look at the whole person, not just what is going on but to see who they are, where they are coming from, their family members and then seeing all that and providing care, centered to the person, to who the person is, because we are all humans, it could just be reversed” (Healthcare Provider, Focus Group #7)*

*“Compassion might be something different for me but I feel that real compassion comes from the heart … compassionate care comes from the heart as opposed to doing what is required … I want to be able to feel genuinely and sincerely and care about people, even the ones that may be difficult” (Healthcare Provider, Focus Group #5)*

This notion of caring for the person behind the disease, was also identified by family members, who felt it was important to remind their HCPs who their loved one was across their lifetime.

*“You have to realize, there’s a person in there … That’s compassion, day-to-day, you know … little things” (Family, Focus Group #1)*

#### Relational communicating

All participants identified that for compassionate care to be authentic there was a need for HCPs to express compassion through verbal and non-verbal communication. Within this population, compassionate communication was inherently relational and corporeal, and was often described as: touching, hugging, crying, being present, sitting, and listening. For residents, family members, HCPs, and mangers alike there was a strong emphasis on the importance of manifesting compassion through tangible behaviors.

*“You don’t have to say a lot of things. Just to be there and know they care enough to be there with you … I can’t say them just be there, give them a hug, hold their hand. That is compassion for me. Words are not really necessary. I don’t think.” (Resident, Focus Group #4)*

*“when someone is dying … , you go close to them, you hold their hand, you listen to them, like when they say can you sit down with me, you say yes I am here for you. For example, that lady, that my friend, when I went there she even told me she loved me, I say I love you too, you know. That is compassion.” (Healthcare Provider, Focus Group #1)*

*“A hug means a lot” (Resident, Focus Group #4)*

While it was never explicitly stated, non-verbal expressions of compassion grew in importance as residents’ cognitive status declined. Further, expressing compassion through non-verbal communication was not described by study participants as being particularly time consuming or grandiose, rather it involved HCPs simply showing residents and their family members their desire to relate to them on a human level. Residents felt that compassion was communicated in little things, such as the tenor of care and the way in which they interacted with them while providing routine care.

#### Attending to needs

The final sub-theme that emerged was *“'attending to needs'”.* Examples and discussions of this came largely from residents and family members who wanted to share personal stories of compassionate acts by their HCPs. Within these stories there was a recognition that compassion often manifested in small acts that were deeply meaningful and special to the individual resident. More these acts attended to needs beyond the resident’s medical needs, extending to acts intended to enhance resident well-being, even if it meant bending the rules or institutional policy.

*“ … you know what truly is the little things … One night my dad decided he wanted a bacon sandwich, the kitchen and everything was closed, … essentially the nurse helped my mom break into the kitchen and let her sit in the and cook up a bacon sandwich for my dad that night and nobody said this is bad idea, no he can’t eat that, none of that kind of stuff. Nobody said there’s rules against that, that was compassionate care because all they cared about was what they little thing that mattered to him at that moment; was there any we could make that happened” (Family, Focus Group #1)*

*“I know one guy who loved his beer and you know an hour before he died he had a sip of beer on a spoon and he died happy you know and so there’s got be compassionate care … looking beyond the rules to what we can we do to make this their home. Turn it from an institution to their home” (Family, Focus Group #3)*

*“I have a night nurse that always, before she goes out of the room, fixes the pillow to make sure I’m comfortable and that one little thing, it just makes me really admire her” (Resident, Focus Group #1)*

### Theme: Organizational Compassion: resources and staffing

The second theme related to contextual factors associated with offering compassion care within LTC. While the previous theme was more theoretical in nature consisting of participant perspectives on the concept of compassion, the theme of *Organizational Compassion: resources and staffing* focused exclusively on the practicalities of how to implement or operationalize compassionate care in LTC. Within this theme there were five subthemes identified: *compassion training*;, *mandated compassion*;, *time*;, *inter-professional compassion*;, and *"death, grief, and mourning*.

#### Compassion training

Across the four groups, there were mixed results about whether someone could be trained in the provision of compassionate care, indicating that compassionate care was also strongly influenced by personal factors such as HCPs innate virtues, life experience, self-motivation, and personal choice. Additionally, those participants who were cynical about the feasibility of compassion training, felt that because compassion was predicated on genuineness, there was a danger that training programs could teach behaviors that family and residents perceive as compassionate that were disingenuous, and antithetical to the nature of compassion itself—a distinction that was described by some focus group members as *being compassionate* versus *doing compassionate care*. These participants felt that an inherent challenge to compassion training programs was cultivating the personal virtues, that were fundamental and a distinguishing feature of compassion, compared to routine caregiving.

*“I think it’s more work experience and I think that’s part of the person. Like some people are more empathetic then others. And some people will never really be compassionate … ” (Healthcare Provider, Focus Group #8)*

*“You can teach people how to handle it but being compassionate is, I think, a matter of the heart” (Healthcare Provider, Focus Group #6)*

*“It’s innate” (Healthcare Provider, Focus Group #4)*

The majority of participants felt that compassion could be trained, however it required a more experiential approach to learning which involved mentorship, self-reflection, and how to communicate compassion in an effective manner. These participants highlighted that like other skills in healthcare, compassionate care can be nurtured, and therefore it needed to be developed and supported through professional training and organizational policy.

*“It can be enhanced for sure... I think with the right character you can teach compassion” (Healthcare Provider, Focus Group #4)*

*“I think we are all in healthcare, because we, I do believe that everybody who is in healthcare does have some form of compassion, we want to be there, we want to care, we want to help but yeah I think you are right, I think there is a need for training” (Manager, Focus Group #2)*

“*Maybe the nurses here have compassionate care, but they don’t know to express themselves. They have not been trained to be compassionate … . They are trained to be nurses.” (Resident, Focus Group #5)*

#### Mandated compassion

Within this subtheme the idea of whether compassion should be mandated for HCPs and LTC was discussed. This subtheme was particularly significant for family members and residents who felt strongly that there was a need to mandate compassion for HCPs and within healthcare systems that they worked. While there was general agreement among HCPs and managers, to make compassionate care an expectation versus an option, residents and family members were adamant that compassion should be monitored and evaluated along with other care outcomes, often citing incidences were compassion was lacking in support of this claim. Of the four participant groups, residents were most vocal about the importance of this theme.

*Well I had one nurse that I had rang the bell and she came and stood at the end of the bed and looked at me and said, “what do you want?” (Resident, Focus Group #1)*

*“Cause you will run into some that really have compassion for a person that is passing away or whatever and there’s some that don’t give a damn” (Resident, Focus Group #2)*

Family members were less explicit about individual HCPs, citing a multitude of challenges that both HCPs and administrators face in providing compassionate care. Nonetheless, they too identified the necessity of mandating compassionate care within healthcare, noting that if it continued to be considered an exception versus an expectation, that HCPs and organizations would not be accountable or motivated to enhance it.

*“Well the thing is, I often think about the institutions and they have a corporate objective, you know? Profit, not for profit, people have performance review, people bring all these things in and the danger is that unless they can keep compassion and compassionate care at the forefront, everyone becomes a widget … .” (Family, Focus Group #5)*

*“You know … I think that is a hard thing and the challenge is that you are still trying to deliver on this compassion, compassionate care in an institutionalized setting and that is tough because they all have a job to do” (Family, Focus Group #2)*

*“I think you can have expectations of staff … the atmosphere towards the resident can be steered by the management … it (compassion) needs to be cultivated, a sense of compassion in a facility and that comes through training, that is not just innate, it is training” (Manager, Focus Group #2),*

HCPs, particularly the registered nurses who worked in managerial positions, also recognized the need for compassionate care to be enhanced, however they were far less reticent to use the language of mandating compassionate care then residents and family members.

#### Time

Time (which was also identified in terms of a lack of staff) was seen to be a chief barrier to providing compassion care across all participant groups. There was nearly unanimous agreement that HCPs were perceived as not having enough time to provide compassionate care in addition to their other duties. This was particularly evident among healthcare aides, who acknowledged feeling ‘stretched’, ‘squeezed’, and frustrated by the inability to have enough time to provide compassionate care to their residents. Several of these participants stated that they would often use their own time, or, creatively adapt their schedule to be able to give the compassionate care that they genuinely desired to give.

*“We use our own time … we squeeze time … . We wish we have more time but we don’t … ” (Healthcare Provider, Focus Group #1)*

*“This is our precious time with this resident. Unfortunately, we should not wait that long [death], but, time is against us” (Healthcare Provider Focus Group, #3)*

*“Limited time” “time” “It is always time” (Manager, Focus Group #2)*

*“They are totally overworked, totally, totally overworked” (Family, Focus Group#1)*

Family members corroborated HCPs concern about the lack of time they had to provide compassionate care, attributing HCPs lack of time, as one of the common contributors of incidences were compassion care was lacking.

#### Inter-professional compassion

In our analysis, inter-professional compassion was defined as the ability for HCPs to offer compassionate care to each other, particularly between different professional groups. Tensions were identified between healthcare aides and registered nurses within HCP focus groups and between HCP focus groups and focus groups comprised of managers. Feeling underappreciated and undervalued by their superiors (healthcare aids feeling undervalued by registered nurses; registered nurses and healthcare aides feeling undervalued by management) was cited as a major impeder of compassionate care, particularly when it involved incidences in which HCPs efforts to provide compassionate care were minimized or ignored. Healthcare aides, in particular, felt that a lack of compassion between professional groups, often made it difficult, and in some incidences hypocritical, to provide compassion to patients.

*“it would be nice if, … the nurses would take your word, that you do know something. You know, we are not just healthcare aides. We are the people, we are the ones that work with these people … . We do know a little bit about what we are talking about” (Healthcare provider, Focus Groups #3)*

*“But if there could be more of a team work effort and you hear that, that is something that bothers me too, you constantly hear from management that there needs to be team work, there needs to be team work but especially in a situation like this, why does everybody’s paperwork come before this person’s life … ” (Healthcare provider, Focus Group #2)*

Interestingly, within the family member focus groups, this subtheme was also acknowledged by family members, who felt that management needed to better acknowledge and appreciate the “hands-on” approach to compassion that they witnessed in their HCPs.

*“But I think it’s also, it would also be really good for the upper level staff to comment to the people who are the hands-on-staff, well you did a really good job there, hey I noticed you did this or man that was a really nice thing you did for that person; to recognize it and that will help to encourage more of that same behavior” (Family, Focus Group #1)*

Management was not ignorant to these issues, acknowledging that dynamics within the care team impeded compassionate care to patients, while also recognizing that they, as managers, needed to model compassionate care to their own employees to enhance compassionate care at the bedside.

*“You have to be compassionate to your staff too cause some people are very afraid of death and if I am aware of that and what not I will ask them to leave the room or to do this or that we will take care of it or whatever” (Manager, Focus Group #1)*

To do this many of the managers, particularly registered nurses who often functioned as clinical managers, felt that they needed to develop an awareness and respect for each individual members’ comfort level related to providing compassionate care.

#### Death, grief & mourning

The final subtheme, within the theme of organizational compassion, focused on the enduring effect that death, grief and mourning had on HCPs, particularly their ability to provide compassionate care. HCPs felt that compassionate care involved forging a meaningful relationship with family and residents, relationships that were severed at the time of death. While participants acknowledged that death and dying are experiences that all HCPs experience to varying degrees, they felt that grief and mourning by HCPs in LTC was complicated by virtue of the fact that their daily interactions with the residents in their care, extended from months to years.

“*Because most of us, we are attached to the resident, because they are here for so long*” *(Healthcare provider, Focus Group #6)*

In anticipation of these losses HCPs expressed a need for support and compassionate care towards them as they worked through their own grief and mourning with each resident's death. This need for compassionate care was identified both in terms of the need for recognition of the relationship that HCPs had established with residents, by affording them the opportunity to say “goodbye”, having time off or a lightened schedule, and public rituals within the facility to help healthcare providers deal with their own grief.

*“I think one of the most important things in my mind is the staff has worked with these residents for a long time, they must be given the opportunity to grieve as well as the family is grieving. I mean it’s a loss. It may not be a family member but it’s someone you may have taken care of for ten years. So, they have to be given the opportunity to grieve” (Healthcare provider, Focus Group #9)*

*“But because the staff have to be given the opportunity to mourn the end too” (Family, Focus Group #1)*

## Discussion

The findings from this study build on a growing body of research illustrating the desire and need to improve compassionate care in healthcare. The qualitative approach provided rich data to explicitly explore the understanding of compassion from not only HCPs, but also importantly, from the perspective of a vulnerable population- residents in LTC and their family members. Results from this study indicate that all participant groups (residents, family members, HCPs, and mangers) viewed compassionate care as an essential component of care within LTC.

The results from this study support the notion that core components of compassion seem to be universally understood, while acknowledging that their expression and experience in practice varies from provider to provider and resident to resident [15, 38, 40]. Through the inductive process of open coding, it became apparent that the core themes identified in this study were congruent with previous compassion models from both the perspective of patients and their healthcare providers [38, 40]. This may be in part due to the fact that the author of these previous models was a member of the coding team, but this potential bias was guarded against by having a third member (LV) of the research team code the data independently and using an audit trail that was further scrutinized by the larger research team. While this potential may not have been completely mitigated, inductive (data generated) and theoretical approaches to analysis (theory generated) are recognized and encouraged in thematic analysis [37]. The research team was somewhat surprised to discover that the themes and sub-themes that emerged from the data were consistent with our previous research in other populations, and are also largely congruent with VanderCingel’s multi-dimensional conceptualization of compassion based on the perspectives of nurses and patients in LTC [36, 38, 40]. This illustrates for us, that compassion and the need for compassionate care cuts across patient populations, disciplines, and care settings. While the core domains of compassion were largely congruent with our previous research, the sub-themes of *seeking to understand* and *relational communicating* seemed to have increased importance within this study population where personhood is perceived as being threatened by cognitive impairment, frailty, and role loss [41]. While these are significant losses that cannot be remedied, they were often allayed through small but meaningful acts such as a supportive touch, listening, and trying to see the person behind the disease.

These results also continue to highlight that compassion is multidimensional and is dependent on a composite of virtues, intentions, relational skills and intentional deliberate actions on the part of the HCP and is less dependent on patient type, patient behavior or features of the resident [36, 42, 43]. Determining that compassion is a combination of the innate qualities and tangible actions of HCPs provides important insight on the ongoing debate within the literature of whether HCPs can be trained in compassion. It would seem that the debate has less to do with the feasibility of compassion training and more to do with need for a multi-pronged pedagogical approach that focuses on cultivating HCPs virtues and equipping them with tangible skills to help express these virtues  in practice.

For all the HCPs who participated in our focus groups, offering compassionate care was considered an essential part of their jobs and a significant source of both personal and job satisfaction. While there was a number of inhibitors to compassion, compassion itself was not one of them, suggesting that providing opportunities for HCPs to provide compassionate care may not only be a buffer against burnout but may have a sustaining effect on HCPs [44]. At the same time, results from this and other studies [45–50], suggest that compassionate care cannot be based on the individual efforts of HCPs, but must be enacted at an organizational and systems level in order to be sustained and optimized. A recent review of the compassion fatigue literature [44] suggested that healthcare providers’ experiences of compassion fatigue are more correctly attributed to other aspects of occupational stress than compassion itself, which was further verified by study participants who spoke about the notion of compassion fatigue sparingly, instead acknowledging healthcare organizations stresses and disenfranchised grief as challenges to compassionate care. Considering this, the institutional obstacles identified in this study that are impeding HCPs availability to provide compassionate care need to be taken seriously, and the question of whether compassionate care should be mandated within LTC facilities warrants further exploration—not only for residents’ well-being but also HCP well-being.

While acknowledging the legitimate impediments to offering compassionate care, these impediments should not be seen as barriers in the strictest sense [51]. Despite the strength of the argument that time was the primary barrier to providing compassionate care, it should also be noted that while “timeliness” was a descriptor of compassion, time itself was not. In addition, all four groups felt that compassion could be imbued through “little things” rather than from any particularly large gesture that required a lot of time. As one resident commented, compassion from a staff member could be as small an act as fluffing his pillow each time before leaving his room. The notion that compassion could be conveyed in small and ‘timeless’ gestures was affirmed by one of the manager participants who felt that compassionate care could be enacted by having her staff say ‘hello’ or intentionally popping in and acknowledging a family member sitting at the beside. While participants did not deny the realities that challenge LTC staffs' ability to be compassionate, including being overworked and chronically short staff, these were not challenges that they felt could not be overcome. In addition, LTC is one of the few healthcare settings where HCPs are afforded a significantly long period of time to develop relationships with their residents. Thus, while “time” on a day-to-day level may be extremely tight, HCPs in LTC facilities do have the advantage that they may have months or years to come to know that individual resident.

The importance of inter-professional compassionate care should be acknowledged. There was a strong sense, especially by HCP participants, that they were undervalued and unsupported by senior staff in their efforts to provide compassionate care [41]. This supports research which highlighted when managers actively worked to fashion a person-centered workplace, it added quality to the life of the caregivers, which then in turn added quality to the life of the residents [52]. This was especially the case in relation to issues of grief and mourning. As developing meaningful relationships with residents is a consequence of providing compassionate care, it is essential that HCPs are given the appropriate supports to address their own needs for grief and mourning. The grief literature has identified that HCPs who are not given appropriate means to fully address their grief are more likely to experience unresolved or complicated grief, both of which have been associated significant physical and mental health complications [53]. The desire of HCPs to have rituals is also supported by grief literature [54, 55], as rituals not only provide a structured time and place to begin the mourning process, but they also create a communal acknowledgement of the grief and mourning that currently is taking place by those who participate. The challenges of death, grief, and mourning, though not explicitly discussed in this study, need to also be acknowledged for family members; particularly as they begin to experience their own sense of loss, grief, and mourning [27].

The results from this study have strong implications for both future research and clinical practice. First, this study gathered rich data from not only HCPs and managers, but also from residents and family members, highlighting not only the importance of compassionate care and related challenges from the perspective of recipients but demonstrating that obtaining residents perspective is not only possible but beneficial. While our findings are from focus groups, and are not generalizable in the same sense as quantitative research, this study validates previous research and models of compassion that may be transferable to other LTC settings [38, 40, 43]. Second, our study also illustrated that further research needs to be done to fully explore the challenges to offering compassionate care in LTC, so that if these are legitimate, changes to support compassionate care at the organizational and policy level can be implemented. For example, greater exploration into inter-professional compassion between professions seems relevant given that healthcare aides often felt that their expressions of compassionate care were minimized or dismissed. Lastly, as a limitation, there is the possibility that our results do not represent the fullness of compassionate care for frail older adults in LTC, because of selection bias since participants were not randomly selected and social desirability bias in terms of their responses—resulting in a sample of individuals who were already interested in the topic and who spoke of the topic, and their relationship to the topic, in favorable terms. Finally, while this large qualitative study revealed a high level of congruence related to the concept of compassion between patients, family members and healthcare providers, in synthesizing the results between these groups, contrasting opinions may have been under reported.

## Conclusion

This study sought to discover the understanding of compassionate care from residents, family members, HCPs and managers within LTC facilities across Canada. All participants within our study expressed a strong desire for compassionate care to be enacted as principal means of providing care in this context. Compassionate care allowed HCPs to look beyond the difficulties associated with caring for frail older adults and to focus on the person and uphold the “humanness” of their residents, which in turn helped to facilitate better quality of life and deaths for residents, and personal meaning and satisfaction for HCPs. Compassion training, mandating compassion, time, inter-professional compassion, and death, grief and mourning arose as potential challenges to HCPs ability to offer compassionate care within LTC facilities. Recognizing and understanding the complexity of factors influencing HCPs ability to offer compassionate care will allow for better research and clinical approaches to high quality patient-centered care.

## Additional file

Additional file 1: Interview Guide. This are the interview guiding questions that were used across all four provinces with each of the different demographic focus groups. (DOCX 51 kb)

## Abbreviations

HCPs: Healthcare Providers
LTC: Long-Term Care
