# Supplementary material for: Perspectives and experiences of compassion in long-term care facilities within Canada: a qualitative study of patients, family members and health care providers
Source: BMC Geriatr. 2019 May 6;19:128. doi: 10.1186/s12877-019-1135-x (PMC6503362; doi:10.1186/s12877-019-1135-x)
Supplement: Supplementary file 1 — Interview Guide. This are the interview guiding questions that were used across all four provinces with each of the different demographic focus groups. (DOCX 51 kb) [file 12877_2019_1135_MOESM1_ESM.docx]

**Supplementary File 1**

**Interview Guiding questions**

1. Can you tell us what compassion means to you? What is compassion in this setting?
2. What impact does compassionate care have on you personally (all participants) and professionally (healthcare staff and managers only)?
3. What are inhibitors of compassion?
4. What are facilitators of compassion?
5. Can you provide examples of compassion in this setting?
6. Do you think you can train someone to be compassionate?
7. To what extent do you think that dementia is a barrier to compassionate care?
8. How important is compassion at end of life? Please explain
